# Different Dopaminergic Dysfunctions Underlying Parkinsonian Akinesia and Tremor

**DOI:** 10.3389/fnins.2019.00550

**Published:** 2019-05-29

**Authors:** Daniele Caligiore, Francesco Mannella, Gianluca Baldassarre

**Affiliations:** National Research Council, Institute of Cognitive Sciences and Technologies, Rome, Italy

**Keywords:** akinesia, resting tremor, Parkinson's disease, phasic and tonic dopamine, dopamine receptors, innovative drug therapies, system-level computational neuroscience, spiking neurons

## Abstract

Although the occurrence of Parkinsonian akinesia and tremor is traditionally associated to dopaminergic degeneration, the multifaceted neural processes that cause these impairments are not fully understood. As a consequence, current dopamine medications cannot be tailored to the specific dysfunctions of patients with the result that generic drug therapies produce different effects on akinesia and tremor. This article proposes a computational model focusing on the role of dopamine impairments in the occurrence of akinesia and resting tremor. The model has three key features, to date never integrated in a single computational system: (a) an architecture constrained on the basis of the relevant known system-level anatomy of the basal ganglia-thalamo-cortical loops; (b) spiking neurons with physiologically-constrained parameters; (c) a detailed simulation of the effects of both phasic and tonic dopamine release. The model exhibits a neural dynamics compatible with that recorded in the brain of primates and humans. Moreover, it suggests that akinesia might involve both tonic and phasic dopamine dysregulations whereas resting tremor might be primarily caused by impairments involving tonic dopamine release and the responsiveness of dopamine receptors. These results could lead to develop new therapies based on a system-level view of the Parkinson's disease and targeting phasic and tonic dopamine in differential ways.

## 1. Introduction

Parkinson's disease (PD) is an heterogeneous neurodegenerative disorder in which patients exhibit different clinical courses and prognoses (Thenganatt and Jankovic, 2014; Mu et al., 2017). Within the PD patient population, it has been proposed that the akinetic and resting tremor subgroups reflect major underlying pathological differences (Fishman, 2008; Zaidel et al., 2009; Moustafa et al., 2016). Akinetic patients typically show temporary episodes of freezing during movement or a difficulty to start voluntary movements, such as walking. In these patients, rigidity may also contribute to a reduced range of movements (e.g., inflexibility of the limbs, neck, or trunk) as the muscles tend to remain stiff and unable to rest (Agid, 1991; Magrinelli et al., 2016). By contrast, people affected by resting tremor exhibit uncontrollable movements that affects a body part, for example the hand, when at rest. Tremor tends to decrease or stop when the patient deliberately moves the affected part of the body (Deuschl et al., 2000; Kalia and Lang, 2015). PD patients with resting tremor can also show akinesia and rigidity, while usually the opposite does not happen. Moreover, they have a better prognosis and slower disease progression than akinetic subtype patients (Berardelli et al., 1983; Zhang et al., 2015).

Several studies have shown that akinesia and resting tremor involve partially different brain circuits and neurobiological processes. Both impairments involve an altered activity in the striato-thalamo-cortical circuit, but akinesia is also associated with changes in the neural activity of the mesolimbic pathway targets (e.g., amygdala) whereas resting tremor is associated with dysfunctions in the cerebello-thalamo-cortical system (Eidelberg et al., 1995; Brown et al., 2001; Mure et al., 2011). Although the occurrence of akinesia and tremor is traditionally associated with the degeneration of the dopaminergic system, the exact nature of the multifaceted impairments it causes is not fully understood (Helmich et al., 2012; Hallett, 2014; Zhang et al., 2015; Karunanayaka et al., 2016). As a consequence, current dopamine medications based on levodopa administration cannot be tailored to the specific dysfunctions of the different patient subtypes. The result is that the administration of the same drug produces different effects, for example akinesia and rigidity usually decrease whereas tremor sometimes persists (Stacy, 2009; Connolly and Lang, 2014).

This article proposes a neurophysiologically plausible computational model to study the role of different dopamine impairments in the occurrence of akinesia and resting tremor. The model has three key features for which it represents a tool for investigating PD in a new way. First, the model architecture is constrained on the basis of the relevant known system-level anatomy of the basal ganglia-thalamo-cortical loops (Alexander, 1986; Gurney et al., 2001, 2004; Caligiore et al., 2017a). This represents a first step toward a more articulated investigation of PD according to a system-level perspective rather than by following a focused approach targeting single brain areas (Caligiore et al., 2016, 2017b; Helmich, 2018). Second, it is implemented with spiking neurons with physiologically-constrained parameters (Brunel, 2000; Humphries et al., 2006). Including this feature is critical to reproduce physiological data in a more realistic way (Maass, 1997; Izhikevich, 2003). Third, the model allows the study of the effects of systematically varying the levels of phasic-dopamine peaks and tonic-dopamine levels and the responsiveness of D2-dopamine receptors within the basal ganglia circuits (Fiore et al., 2014; Mannella and Baldassarre, 2015). On this basis, the model is able to reproduce the recently discovered neural mechanism for which the triggering of a movement requires a dopaminergic burst just before the movement onset (Jin and Costa, 2010; Howe and Dombeck, 2016; da Silva et al., 2018) as well as the mechanisms underlying the critical role of D2 receptors in the regulation of the dopamine release in PD (Bolan et al., 2007;Hisahara and Shimohama, 2011).

The computer simulations run with the model show how all these features, to date never integrated within a single computational model, are critical to understand the neural mechanisms underlying the occurrence of akinesia and resting tremor. In more detail, the model suggests that akinesia and resting tremor are caused by different dopaminergic dysfunctions. Akinesia can be caused by phasic dopamine dysregulations (simulated with a reduction of the dopaminergic burst and with a desynchronization of the burst occurrence time with respect to the movement onset) as well as by tonic dopamine impairments (simulated with a reduction of the dopamine baseline level). Instead resting tremor is mainly due to tonic dopamine dysfunctions and the reduced activation of D2-dopamine receptors. Aside from this, the model also confirms the main results obtained in the experiments with primates (e.g., Bergman et al., 1994) and humans (e.g., Rodriguez-Oroz et al., 2001; Heida et al., 2013) concerning the neural discharge of subthalamic nucleus neurons during parkinsonian tremor. Overall, these results enhance our understanding of the basal ganglia-thalamo-cortical circuit physiology and could be important to develop new therapies for PD following a system-level perspective and based on an independent manipulation of phasic and tonic dopamine (Caligiore et al., 2016, 2017b).

The rest of the paper is organized as follows: section 2 describes the computational details of the model and the biological support of its assumptions. Section 3 presents the results and the predictions obtained with the model. Section 4 discusses the system-level mechanisms and the different dopaminergic manipulations through which the model explains the occurrence of akinesia and tremor. This section also compares the model with other similar computational systems proposed in the literature. Finally, section 5 presents some limitations of the model and suggests possible future work to overcome them.

## 2. Methods

### 2.1. Model of Single Neurons

The model was built using the *Neuron Simulation Tool—NEST* (Gewaltig and Diesmann, 2007). In particular, we used *PyNEST* (Eppler et al., 2009), the *Python*^1^ programming language interface of *NEST*. The simulations were run through the *Grid'5000* high-performance computing facility (Balouek et al., 2013). Within the model, each neuron is modeled as a leaky integrate-and-fire unit with exponential shaped post-synaptic currents. A spike is modeled with an infinitely short-time current peak and is generated when the membrane potential *V*_*m*_ reaches the threshold value *V*_*th*_. The spike is sent with delay *t*_*delay*_ to all post-synaptic neurons. The threshold crossing is followed by an absolute refractory period *t*_*ref*_ during which the membrane potential is clamped to the resting potential *V*_*reset*_ and spiking is prevented. Mathematically, the dynamics of the neuron membrane potential *V*_*m*_ is given by:

(1) $$\begin{array}{l} \tau_{m}{\dot{V}}_{m}=-V_{m}+R\cdot I \end{array}$$

where τ_*m*_ is the membrane time constant, *R* is the neuron input resistance, and *I* is the sum of various current inputs modeling the post-synaptic current contributions made to the membrane potential by synaptic events. The dynamics of *I* is described by some equations discussed in detail in Tsodyks et al. (2000), Humphries et al. (2006), and omitted here for brevity. The neuron dynamics is numerically integrated based on a computation time step of 1 *ms* and all incoming and emitted spikes are forced to happen in the resulting time grid steps.

To implement this spiking neuron model we used the NEST function *iaf_psc_exp* having the following parameters: membrane potential (*V*_*m*_); spike threshold (*V*_*th*_); resting membrane potential after a spike (*V*_*reset*_); constant input current (*I*_*e*_); resting membrane potential (*E*_*L*_); capacity of the membrane (*C*_*m*_); membrane time constant (τ_*m*_); time constant of post-synaptic excitatory currents (τ_*syn*_*ex*_); time constant of post-synaptic inhibitory currents (τ_*syn*_*in*_); duration of refractory period (*t*_*ref*_); point in time of last spike (*t*_*spike*_). The values of most of these parameters are summarized in Tables 1–3. When possible, the anatomical and physiological data used to set the values of these parameters were derived from works with primates or humans, or from studies with murine models (the tables give information on this). For the parameters not showed in the tables, we used the default values of the *NEST* neuron model *iaf_psc_exp*. Importantly, the different features of PD related to akinesia and tremor were obtained through different damages of the same model with same parameters, an important prerequisite to arrive to the identification of the system-level mechanisms underlying those features.

**Table 1:** BG parameters.

**Area**	**Parameter**	**Source**
Striatum-D1/D2	τ_*m*_ = 25.0 *ms*	Beninger and Olmstead, 2000
	τ_*ref*_ = 2.0 *ms*	
	*C*_*m*_ = $\frac{\tau }{R}\;pF$	
	*R* = 42.0 *M*	Jiang and North, 1991; Flores-Hernández et al., 1997
	*V*_*th*_ = 30.0 *mV*	
	*V*_*reset*_ = −20.0 *mV*	
	*I*_*e*_ = 0.0 *pA*	
STN	τ_*m*_ = 6.0 *ms*	Kita et al., 1983; Paz, 2005
	τ_*ref*_ = 2.0 *ms*	
	$C_{m}=\frac{\tau }{R}\;pF$	
	*R* = 18.0 *M*	Kita et al., 1983
	*V*_*th*_ = 20.0 *mV*	Beurrier et al., 1999, 2000
	*V*_*reset*_ = −20.0 *mV*	
	*I*_*e*_ = 1000.0 *pA*	
GPe	τ_*m*_ = 18.0 *ms*	Kita and Kitai, 1991
	τ_*ref*_ = 2.0 *ms*	
	$C_{m}=\frac{\tau }{R}\;pF$	
	*R* = 88.0 *M*	Kita and Kitai, 1991
	*V*_*th*_ = 30.0 *mV*	Cooper and Stanford, 2000
	*V*_*reset*_ = −20.0 *mV*	
	*I*_*e*_ = 350.0 *pA*	
GPi	τ_*m*_ = 8.0 *ms*	Nakanishi et al., 1987, 1997
	τ_*ref*_ = 2.0 *ms*	
	$C_{m}=\frac{\tau }{R}\;pF$	
	*R* = 112.0 *M*	Nakanishi et al., 1987, 1997
	*V*_*th*_ = 30.0 *mV*	Atherton, 2005
	*V*_*reset*_ = −20.0 *mV*	
	*I*_*e*_ = 200.0 *pA*	

**Table 2:** M1 and Thal parameters.

**Area**	**Parameter**
Thal	τ_*m*_ = 20.0 *ms*
	τ_*ref*_ = 2.0 *ms*
	*C*_*m*_ = 1.0 *pF*
	*V*_*th*_ = 30.0 *mV*
	*V*_*reset*_ = 4.0 *mV*
	*I*_*e*_ = 4.0 *pA*
M1	τ_*m*_ = 20.0 *ms*
	τ_*ref*_ = 2.0 *ms*
	*C*_*m*_ = 1.0 *pF*
	*V*_*th*_ = 30 *mV*
	*V*_*reset*_ = 10.0 *mV*
	*I*_*e*_ = 0.0 *pA*
	*N*_*e*_ = 800
	*N*_*i*_ = 200
	*W*_*e*_ = *N*_*e*_/100.0
	*W*_*i*_ = *N*_*i*_/4.0

*The values of the parameters for M1 were drawn from Brunel (2000)*.

**Table 3:** Connection parameters.

**Connection**	**Parameter**	**Type**
Cortical input → StrD1	*t*_*d*_ = 1.0 *ms*	C2C
	*w* = 50.0	
	ρ = 0.25	
Cortical input → StrD2	*t*_*d*_ = 1.0 *ms*	C2C
	*w* = 50.0	
	ρ = 0.25	
Cortical input → STN	*t*_*d*_ = 2.5 *ms*	C2C
	*w* = 50000.0	
	ρ = 0.25	
StrD1 → GPi	*t*_*d*_ = 4.0 *ms*	C2C
	*w* = −1200.0	
	ρ = 0.25	
StrD2 → GPe	*t*_*d*_ = 5.0 *ms*	C2C
	*w* = −1000.0	
	ρ = 0.25	
STN → GPe	*t*_*d*_ = 2.0 *ms*	A2A
	*w* = 400.0	
	ρ = 0.08	
STN → GPi	*t*_*d*_ = 1.5 *ms*	A2A
	*w* = 500.0	
	ρ = 0.08	
GPi → Thal	*t*_*d*_ = 3.0 *ms*	C2C
	*w* = −100	
	ρ = 0.25	
GPi → GPi	*t*_*d*_ = 1.0 *ms*	C2C
	*w* = −100	
	ρ = 0.25	
GPe → STN	*t*_*d*_ = 4 *ms*	A2A
	*w* = −300	
	ρ = 0.25	
GPe → GPi	*t*_*d*_ = 3 *ms*	A2A
	*w* = −20	
	ρ = 0.25	
Thal → M1	*t*_*d*_ = 1.0 *ms*	A2A
	*w* = 60.0	
	ρ = 0.5	
M1 → Thal	*t*_*d*_ = 1.0 *ms*	C2C
	*w* = 50.0	
	ρ = 0.5	
M1 → StrD1	*t*_*d*_ = 2.0 *ms*	C2C
	*w* = 50.0	
	ρ = 0.5	
M1 → StrD2	*t*_*d*_ = 2.0 *ms*	C2C
	*w* = 10.0	
	ρ = 0.25	
M1 → STN	*t*_*d*_ = 2.0 *ms*	C2C
	*w* = 10000.0	
	ρ = 0.25	

*t_d_ is the connection delay, w is the connection weight, ρ is the proportion of connections that are not set to zero*.

A2A stands for “all-to-all,” C2C stands for “all-to-all within channel.”

### 2.2. Model Architecture and Functioning

**Figure 1:**
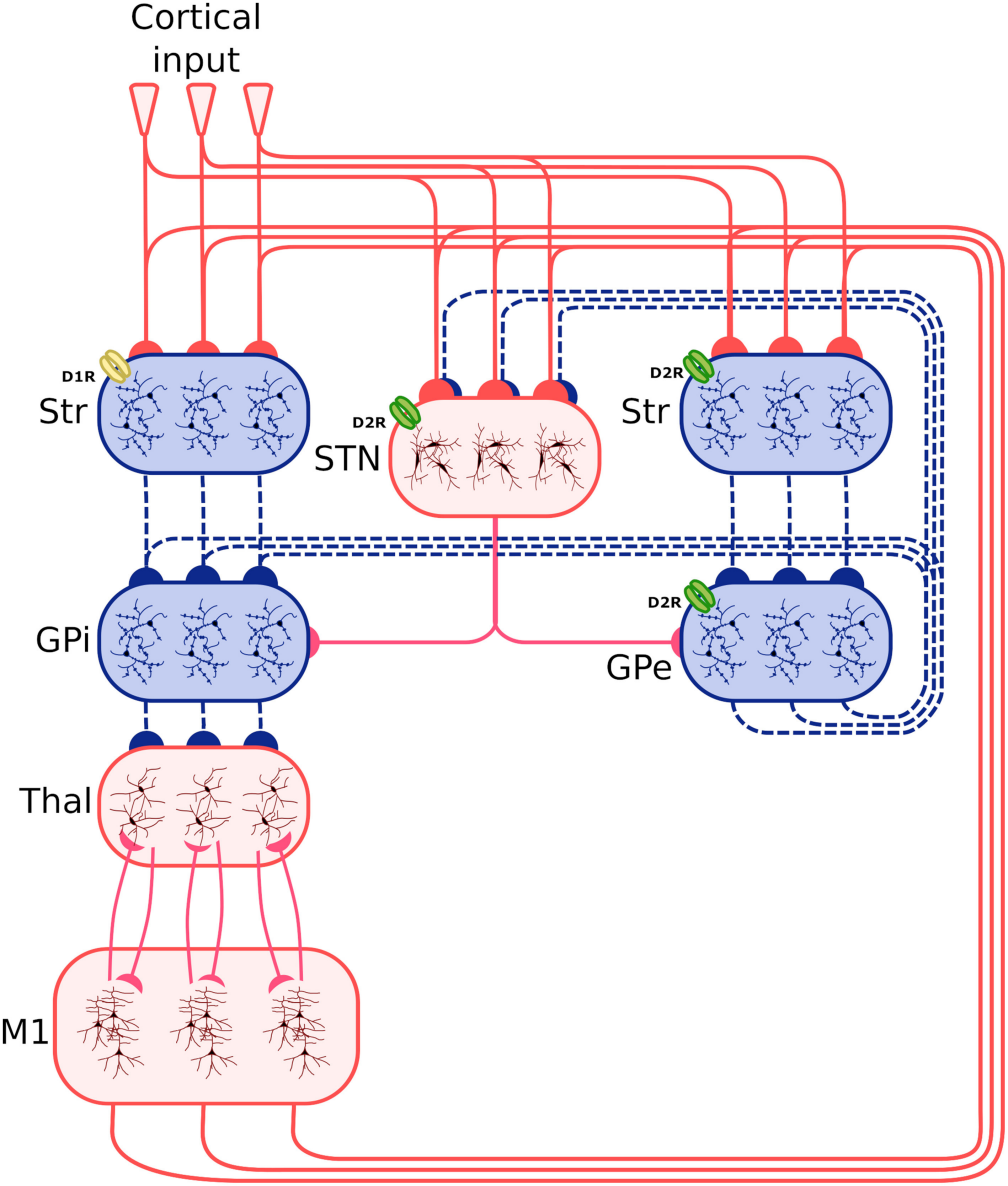
Architecture of the spiking-neuron system-level model. The blue boxes indicate areas projecting through inhibitory connections (dashed blue lines), whereas the red boxes indicate areas projecting through excitatory connections (continuous red line). The three lines connecting the boxes, and the three neuron subpopulations within each component, indicate that the synapses between different components is organized through three cortico-striatal-thalamo-cortical channels. The bottom larger box represents the two excitatory and inhibitory neural populations forming the primary motor cortex component (M1). Other abbreviations: StrD1, D1R-expressing striatal populations; StrD2, D2 Receptor (D2R)-expressing striatal populations, STN, subthalamic nucleus; GPe, external globus pallidus; GPi, internal globus pallidus; Thal, thalamus; Cortical input: Poisson generators simulating the effects of other cortical regions on the system. D1R and D2R dopamine receptors, highlighted respectively with a yellow and a green color, have respectively an excitatory and an inhibitory effect. Note how the D2R receptors also act on the STN and the GPe.

Figure 1 shows the system-level architecture of the model. This is formed by eight neural populations of spiking neurons, connected through excitatory and inhibitory connection weights carrying signals modulated by dopamine (see section 2.4).

These neural populations can be clustered in three groups: the BG, capturing the key anatomical and functional features of the basal ganglia; the Thal, reproducing critical anatomical and physiological features of motor thalamus; and M1, reproducing the primary motor cortex. The architecture of BG builds on the spiking-neuron model proposed in Humphries et al. (2006). This is formed by five neural populations representing different sub-regions of the BG. Thal is formed by a single neural population. M1 is formed by two neural populations of respectively excitatory and inhibitory units. This component is implemented building on the spiking-neuron model proposed in Brunel (2000). The model architecture is organized in three cortico-striatal-thalamo-cortical loops, with 64 neurons per channel, making a total of 192 neurons per population. This organization agrees with data supporting the organization of the BG connectivity through parallel anatomical loops running throughout the cortico-striatal-thalamo-cortical system. According to this view, loops form closed circuits running in parallel, each of which originates from a specific cortical area, for example the M1, passes through the BG, and returns to the originating cortical area via the Thal (Alexander, 1986; Middleton and Strick, 2000). In addition to this, different regions of striatum also receive input from out-of-loop different cortical areas (Romanelli et al., 2005). These loops are () proposed to be internally organized in partially segregated channels able to select different cortical contents, for example actions or thoughts (Mink and Thach, 1993; Redgrave et al., 1999). In the model, this anatomical pattern is reproduced through connections linking the subpopulations within each channel, and no connections between channels (with few exceptions, the most notable one being the subthalamic nucleus (STN) that is connected to all subpopulations of its target regions).

Based on this anatomical organization, the model functioning follows current views of the cortico-striatal-thalamo-cortical dynamics (Bolam et al., 2000; Middleton and Strick, 2000; Baldassarre et al., 2013; Caligiore et al., 2013). The striatum is the principal input nucleus of the BG, receiving signals from the Thal (Smith et al., 2004), and the M1 (Glynn and Ahmad, 2002). Most projection neurons of the striatum cells are GABAergic (Redgrave et al., 1999). The model reproduces the partition of the striatum into two projection neuron populations (StrD1, StrD2) based on their dominant dopamine receptor type (D1 or D2 type) (Bolam et al., 2000; Mannella and Baldassarre, 2015). Some data support the co-localization of these receptors in some of the projecting neurons (Surmeier et al., 1997). However, converging evidence suggests a functional segregation between D1- and D2-dominant projection neurons and, furthermore, that the D1-dominant neurons tend to project to the output nuclei of the BG—the internal globus pallidus (GPi) and the substantia nigra pars reticulata (SNr) (in the model we considered only GPi projecting to M1), whereas the D2-dominant neurons tend to project internally to the BG—in particular to the external globus pallidus (GPe) (Gonon, 1997; O'Connor, 1998; West and Grace, 2002; Sano et al., 2003). The STN forms the other primary input nucleus of the BG receiving signals from the M1 alongside the GPe. The neurons of the STN project to target areas through glutamatergic connections (Temel et al., 2005). Both striatal and subthalamic projection neurons send axons to the GPe and the GPi (Bolam et al., 2000). The GPe neurons are GABAergic and project to GPi and STN (forming a loop with this area) (Smith et al., 1998). The GABAergic cells of the BG output nuclei contact numerous Thal nuclei (Bolam et al., 2000).

The M1 component consists of two neuron populations: a population of *N*_*e*_ excitatory neurons (80% of the total M1 neurons) and a population of *N*_*i*_ inhibitory neurons. Incoming excitatory and inhibitory spikes affect the membrane potential *V*_*m*_ by respectively *W*_*e*_ and *W*_*i*_, the strengths of the excitatory and inhibitory connection weights. The neurons are mutually connected with a probability of 10%, thus each neuron receives input from about 0.1 × *N*_*e*_ excitatory and 0.1 × *N*_*i*_ inhibitory neurons of its own channel. The inhibitory synaptic weights *W*_*i*_ are chosen with respect to the excitatory synaptic weights *W*_*e*_ such that *W*_*i*_ = −5.0 × *W*_*e*_. In addition to the sparse recurrent inputs from within the local channel subpopulation, the three channel excitatory/inhibitory subpopulations of M1 neurons receive excitatory input from the corresponding channels of the Thal and send excitatory input to the three subpopulations of Thal, StrD1, StrD2, and STN. The values of the parameters of the BG, Thal, and M1 are summarized in Tables 1, 2, whereas the values of the connection parameters are shown in Table 3.

The amount of each channel activation at the level of the striatum represents the *salience* of the action, causing a certain probability that the action is selected and performed (Redgrave et al., 1999; Hikosaka et al., 2000). The model reproduces the mechanisms underlying the focused tonic inhibition and temporary release exerted by the output nuclei of the BG onto the Thal. The temporary disinhibition, triggering the selection of the channel Thal-M1 subpopulations, can be caused by the focussed activation of the StrD1 projection neurons that is superimposed to the diffused excitation received from the STN (Chevalier and Deniau, 1990). At the same time, the diffuse excitation of the STN could increase the tonic inhibitory output of the other output channels, enhancing the contrast between selected and non-selected channels (Mink and Thach, 1993; Gurney et al., 2001; Nambu et al., 2002). To achieve the correct balance between excitation and inhibition in this circuit, excitation from the STN needs to be regulated by the inhibition from GPe. The circuit comprising StrD2 projection neurons, STN, and GPe provides the needed amount of inhibition required to enable channel selection and switching (Gurney et al., 2001).

### 2.3. Cortical Input

The input to the model coming from other cortical regions (“Cortical input”) was simulated as spike trains generated through a Poisson process (linked to the temporal quantization determined by the simulation time step *t*) having a given frequency rate expressed in spikes per seconds (*sp*/*s*). This assumption agrees with empirical evidence and models showing that the temporal distribution of cortical spikes can be approximated through Poisson processes (Dayan and Abbott, 2001). The Cortical input source was simulated with the *NEST* function *poisson_generator* having the following parameters: mean firing rate (*rate*); time origin of the simulation (*origin*); beginning of device application with respect to origin (*start*); termination of device application with respect to origin (*stop*). The values of the Cortical input source are shown in Table 4.

**Table 4:** Parameters of the Poisson generators simulating the “Cortical input.”

**Channel**	**Parameter**
Channel 1	*Rate* = 20.0 Hz
	*Origin* = 0.0 ms
	*Start* = 5, 000.0 ms
	*Stop* = 7, 999.0 ms
Channel 2	*Rate* = 25.0 Hz
	*Origin* = 0.0 ms
	*Start* = 6, 000.0 ms
	*Stop* = 7, 999.0 ms

*Impulses are fed to Channel 1 and Channel 2*.

### 2.4. Dopamine Modulation

The model reproduces the effects of phasic and tonic dopamine manipulations and the roles played by the different receptors D1R and D2R on the synaptic transmission of striatum, STN, and GPe. In this respect, empirical evidence shows that relevant changes of the coupling of these nuclei are caused by experimental manipulations altering phasic and tonic dopamine (Schultz, 1998; Magill et al., 2001; Baufreton, 2005; Dommett et al., 2005; Bolan et al., 2007; da Silva et al., 2018). Importantly, here we focused on modeling and studying the effects of tonic and phasic dopamine on the target systems while we did not investigate the mechanisms producing them. To simulate the effects of dopamine, we built a new *NEST* module called *modmodule*^2^ implementing in a biologically plausible way the dopamine neuromodulatory effect on synaptic efficacy. This module in particular provides a means to modulate the synaptic conductivity (weights of connections) of connections linking two neural populations based on the activity of a third dopaminergic population. This activity was obtained through a Poisson generator whose parameters are shown in Table 5, together with those of the *modmodule*. The new module adds two types of modulated synapses to the *NEST* simulator. The first, called “d1_synapse,” reproduces the functions of D1R and provides a way to multiply the baseline value of the connection weight with the rate of spikes coming from the dopaminergic population:

(2) $$\begin{array}{r} w_{m}=w\left(1+\alpha_{1}\cdot DA\right) \end{array}$$

where *w* is the weight baseline value, *w*_*m*_ is the weight of the connection including the modulatory effect, *DA* is the rate of the dopaminergic spikes, and α_1_ is a parameter reproducing the effect of the D1R responsiveness to the dopaminergic modulation. This synapse can be used to reproduce multiplicative excitatory modulation as described in Humphries et al. (2006) and Fiore et al. (2014, 2015). The second, called “d2_div_synapse,” reproduces the functions of D2R and provides a way to divide the baseline value of the connection weight for the rate of spikes coming from the dopaminergic population:

(3) $$\begin{array}{r} w_{m}=\frac{w}{1+\alpha_{2}\cdot DA} \end{array}$$

where α_2_ is a parameter defining the D2R responsiveness to the dopaminergic modulation. This synapse can be used to reproduce inhibitory modulation as described in Fiore et al. (2014, 2015). Changing the values of *DA* can be used to manipulate the phasic and tonic features of the dopamine signal (see section 3).

**Table 5:** Parameters of the dopamine system used to simulate healthy individuals and patients with dopamine damages, and parameters of the Poisson generator.

**Receptor type**	**Value**
*D*1*R*_*Str*_	α_*D*1_ = 50
*D*2*R*_*Str*_	α_*D*2_ = 50
*D*2*R*_*GPe*_	α_*D*2_ = 5,000 (2,000)
*D*2*R*_*STN*_	α_*D*2_ = 5,000 (2,000)
DA baseline rate	= 75 Hz (46 Hz)
DA peak rate	= 195 Hz (130 Hz)
DA peak positions =	4,995 ms
	5,995 ms (5,895 ms)
	6,995 ms

*The values used to simulate patients are shown in brackets*.

## 3. Results

This section presents the results obtained by testing five versions of the same model. The first version involves an undamaged dopamine system and is used to simulate healthy individuals. The other four versions of the model are used to simulate PD patients affected by four different impairments of the dopaminergic system: (a) a reduced synchronization between the dopaminergic phasic burst and the movement onset; (b) a reduced peak of the dopaminergic phasic peak; (c) a reduced tonic dopamine baseline level; (d) a reduced responsiveness of dopamine D2 receptors. The values of the parameters used to simulate healthy individuals and PD patients are summarized in Table 5.

The section shows the following data related to the model functioning: (a) for both healthy individuals and patients, the neural activity (average firing rate) of each component of the model during the generation of voluntary movements; (b) for both healthy individuals and patients, the neural activity of STN at rest (i.e., with no input supplied to the model); (c) the STN neurons oscillatory frequency and the action selection efficacy of the system as a function of different dopamine baseline levels and of different D2 receptors responsiveness. The data (b) and (c) are useful to empirically validate the model since they confirm the main results obtained in experiments with primates (e.g., Bergman et al., 1994) and humans (e.g., Rodriguez-Oroz et al., 2001; Heida et al., 2013) about neural discharge of STN neurons in PD patients. The data (a) and part of (c) represent predictions of the model that are also potentially relevant for therapy.

The intention to produce a voluntary movement was simulated by pivoting on the action selection mechanism operating within the cortico-striatal-thalamo-cortical system (Gurney et al., 2001; Baldassarre et al., 2013). In particular we stimulated two channels of the model with two different Cortical input signals. These signals represent the effect of the projections that originate from cortices outside the loop with motor cortex and reach the motor loop striatum portion, and differentially bias the activation of the channels. We left the third channel without stimulation for control. The simulated individual is assumed to perform a voluntary movement when the Cortical input signal in one channel produces, through the cortico-striatal-thalamo-cortical loops, a strong activation of the corresponding neural subpopulation within M1.

### 3.1. Action Selection in Simulated Healthy Individuals

**Figure 2:**
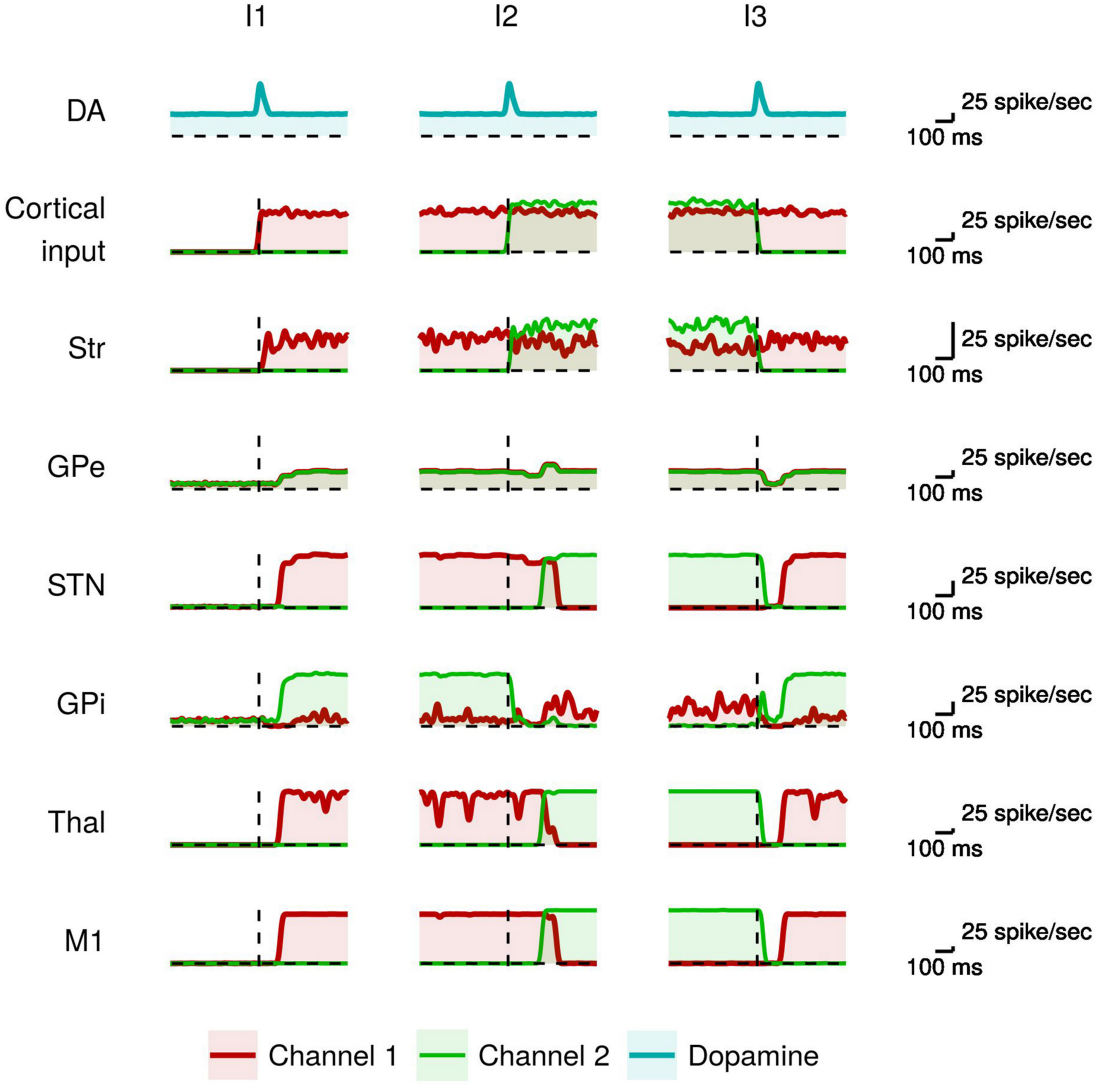
Activity in the healthy model during action selection. From top to bottom: the dopaminergic signal (DA); the input signals activating the channel 1 (red) and the channel 2 (green) from other cortices; mean instantaneous firing rate across all neurons in a particular channel of each region of the basal ganglia (Str, GPe, STN, GPi), thalamus (Thal), and primary motor cortex (M1). The dashed vertical line indicates the onset of the dopamine signal.

We first tested the ability of the model to produce voluntary movements as in healthy individuals (undamaged dopamine system). The simulation we ran to test this condition took 3 s in total and used a stimulus protocol based on 3 succeeding time intervals, denoted as *I*1, *I*2, and *I*3, respectively, having the following elements. Channel 1 is highly activated during all the three intervals. Channel 2 is activated during *I*2, with an activation slightly higher than channel 1, and is not activated during *I*1 and *I*2. Channel 3, used for control, is not activated in any interval (for simplicity we do not report the data related to this channel: its neural subpopulations activate as those of the other channels when they receive no input signal). This sequence of input signals tests the ability of the BG to select a movement within channel 1 (*I*1), then to switch to the more salient movement triggered by the input signal sent to channel 2 (*I*2), and finally to switch again to the initial movement of channel 1 (*I*3). Figure 2 shows the activity of the neural subpopulations of the healthy model caused by the input sequence.

The presence of a salient input is necessary but not sufficient to select a movement. In particular it requires to be coupled with proper values of tonic and phasic dopamine (Humphries et al., 2006; Mannella and Baldassarre, 2015). Moreover, it requires a proper timing of the occurrence of the dopaminergic phasic burst that should happen just before the voluntary trigger of the movement (da Silva et al., 2018). In the model, during *I*1 there is a dopaminergic burst just before the onset of the salient input that is sent to channel 1 and represents the intention to trigger the related movement. When these two events co-occur and have a sufficient intensity the consequent strong striatal activity makes the GPi neurons of channel 1 highly inhibited. At the same time, the neighboring GPi neural subpopulations of the other channels are excited by the STN glutamatergic projections. As a consequence, the output of the GPi subpopulation of channel 1 falls below its initial baseline level (red line), whereas the output of the GPi subpopulation of channel 2 increases above the baseline (green line). This leads to a selective disinhibition of the channel 1 thalamo-cortical loop, causing the activity of the corresponding neurons within M1. The cortical feedback projections from M1 to StrD1, StrD2, and STN stabilize the selection through a cumulative dynamical process (Fiore et al., 2014; Mannella and Baldassarre, 2015).

Subsequently (*I*2), there is a new dopaminergic burst immediately followed by the onset of a stronger input to channel 2. Channel 2 striatal activation causes the inhibition of the related GPi neural subpopulation (green line). This is followed by a short time interval during which the GPi neurons of channels 1 and 2 exhibit low activity and the system cannot yet select the new motor program. The occurrence of the dopamine signal supports the inhibition of the GPe neural subpopulations in both channels (cf. Equation 3). This GPe lower activity, together with the stronger channel 2 striatal signal, causes an increase of the GPi signal in channel 1 (red line). This dynamics supports a selective disinhibition of the channel 2 thalamo-cortical loop in turn causing the activity of the corresponding neurons in M1 (green line). The greater M1 activity in channel 2 propagates to STN that further contributes to strengthen the selection process. In particular, while the STN glutamatergic excitation of the GPi subpopulation of channel 2 is compensated by the high inhibition from the striatum, the other GPi subpopulations are only excited by the STN projections and thus inhibit their target Thal-M1 regions. The increased STN activity also contributes to excite the GPe neural populations that in turn inhibit the STN activity, thus forming a negative feedback loop (cf. section 2).

When the more salient input signal is again the one of channel 1 (*I*3), the selection process works similarly to what happened in *I*2 but with switched roles played by channel 1 and channel 2. Overall, in the healthy individual the output signals of BG correctly select the most salient action and switch to other actions when these become more salient.

### 3.2. Simulated Patients With Dopaminergic Dysfunctions

In order to characterize different PD patients subtypes (in particular akinetic vs. tremor patients), we tested the ability of simulated PD patients affected by different dopaminergic dysfunctions to produce voluntary movements. To this purpose, we focused on the behavior of the model during the challenging time interval *I*2 when there are two competing Cortical input signals with slightly different input signals.

#### 3.2.1. Simulated Phasic Dopamine Dysfunctions Cause Akinesia During Movement Sequencing

**Figure 3:**
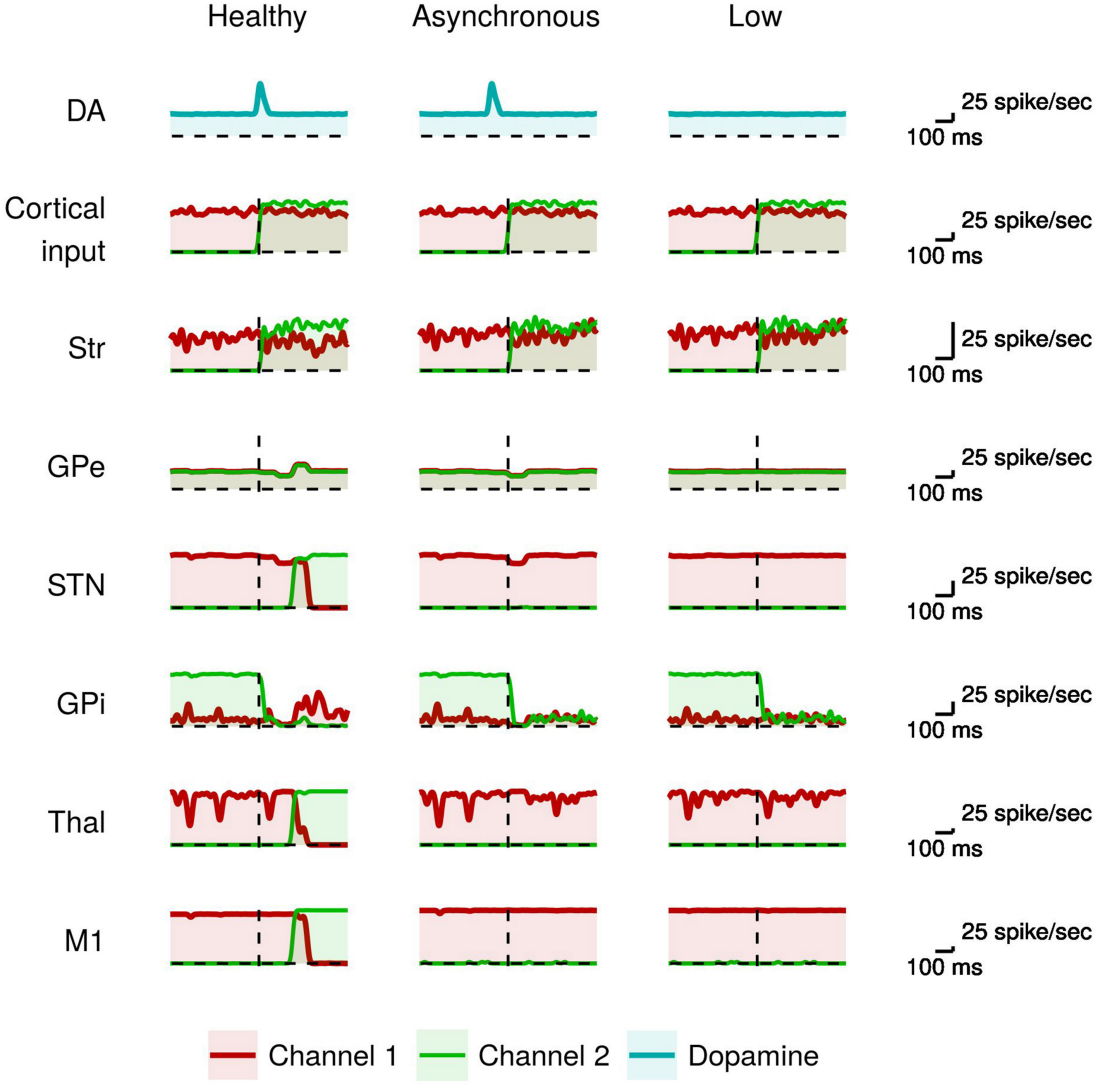
Activity of neural subpopulations in the healthy model vs. the model with phasic dopamine impairments during *I*2. From top to bottom: same data as those illustrated in Figure 2. From left to right: data collected in the model with no dopamine impairments (Normal); in the model with a reduced synchronization between the dopamine burst and the movement onset (Asynchronous); and in the model with a reduced peak of the dopamine burst (Low).

Figure 3 compares the neural activity exhibited by the healthy model with those of the model having phasic dopamine dysfunctions.

In the healthy model, the proper dopamine level in the presence of a high input activation leads to a high striatal activity of the neurons of channel 2 with respect to those of channel 1 (green line). As discussed above, this stronger activity, together with the involvement of the GPe/STN loop, are crucial to trigger the action selection mechanism. By contrast, in the case of dopamine impairments the more salient input to channel 2 alone is not sufficient to start a differential dynamics between the two channels (cf. Equation 2). In particular, the dopamine impairments could lead to a too early occurrence (Asynchronous case), or a too low value (Low case), of the inhibitory effect on GPe that thus fails to cause, via the STN, the “reset” of the system paving the way to the selection of the new movement (cf. Equation 3). As a consequence, the system remains “locked in” the channel 1 activation (red line), so failing to amplify the small difference between channel 1 and channel 2 activations needed to initiate the new movement. These results suggest that dysfunctions of phasic dopamine release could characterize akinetic patients that tend to remain frozen in the activation of particular muscle synergies, in particular to be incapable of switching between two sequential movements. The activity of the channel 1 M1 neurons (red line) during I2 indicates that the model has performed the movement in I1 correctly. That is, the model was able to initiate the first movement when starting from a resting state even if there is a phasic domamine dysregulations. In the next section, we will see that to produce akinesia in this case it is necessary to have a dysregulation of the tonic dopamine release.

#### 3.2.2. Simulated Tonic Dopamine Dysfunctions Cause Tremor and Akinesia From Resting Position

**Figure 4:**
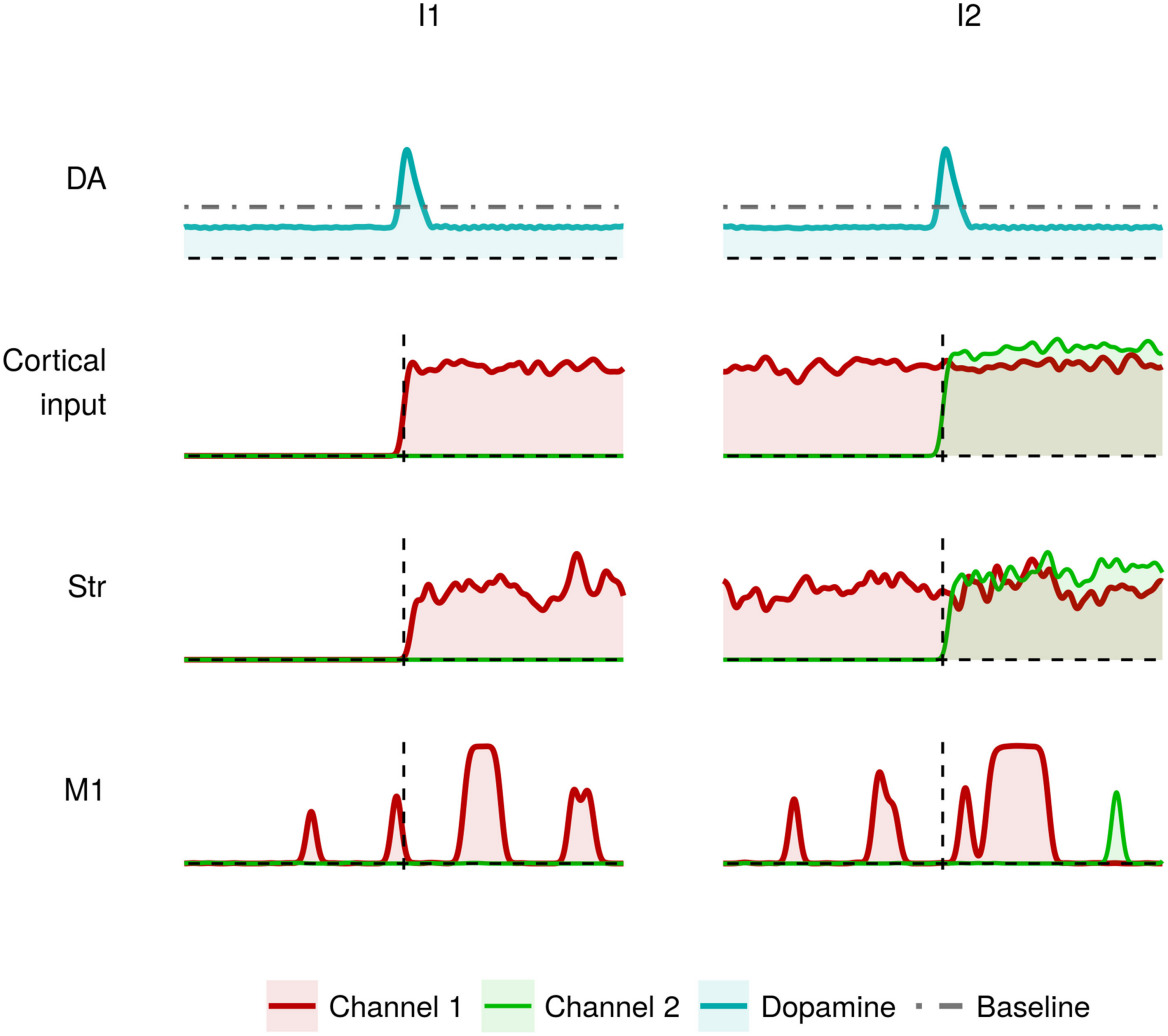
Activity of the critical areas of the model affected by tonic dopamine impairments (lower baseline; lower dopamine D2 receptor responsiveness) in correspondence to heavily (I1) and slightly (I2) differentiated activation of channel 1 and channel 2.

Figure 4 shows the effects of dysfunctions involving tonic dopamine regulation, in particular related to a reduced baseline level and to a diminished responsiveness of the dopamine D2 receptors (α_2_ parameter in Equation 2). In this case, the action selection mechanism works in a sub-optimal way. During I1, the channel 1 M1 neurons result partially activated (red line). As a result, the system finds it difficult to produce a voluntary movement starting from a resting condition. Similarly, the channel 2 M1 neurons result partially activated (green line) meaning that the system is able to start a voluntary movement but with less accuracy and vigor (Mazzoni et al., 2007; Niv et al., 2007). This behavior could be exhibited by tremor patients showing akinesia when starting a simple movement.

**Figure 5:**
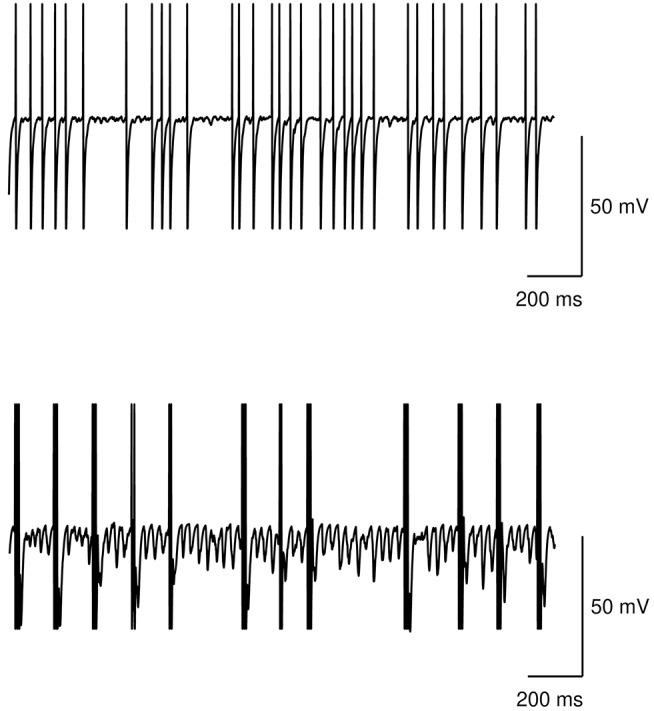
Action potential at rest of the STN subpopulation. Top: healthy model. Bottom: model with tonic dopamine impairments (low baseline level and low dopamine D2 receptor responsiveness).

Figure 5 shows the neural activity of the STN subpopulation with the model at rest, i.e., with no inputs, in the case of no dopamine impairments and the case with tonic dopamine dysfunctions. The figure show that while the healthy model exhibits a random baseline activation, the impaired model exhibits clustered spike trains that repeat at regular times and can be considered the neural correlate of tremor. These results agree with typical data collected in real patients (e.g., Rodriguez-Oroz et al., 2001).

**Figure 6:**
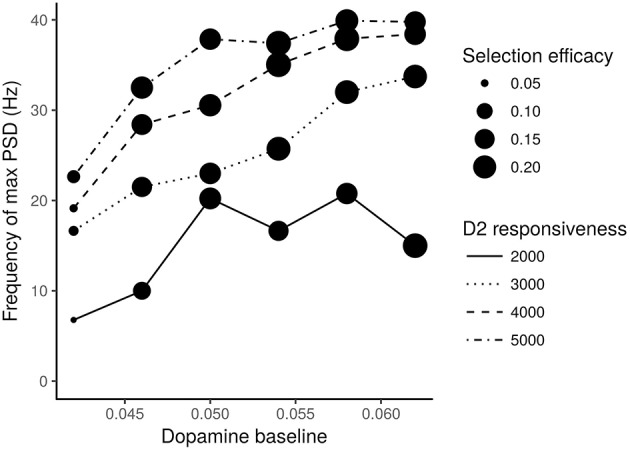
STN neurons frequency of max PSD vs. different levels of dopamine baseline. Different line traits indicate different values of the D2 receptors responsiveness. Expected dots with different size indicate different selection efficacies of the action selection process. The numbers next to the dots represent the mean square errors computed according to a statistics which takes into account the M1 units activated in correspondence to the most salient input.

We also tested the effects of different values of dopamine baseline and of different dopamine D2 receptors responsiveness produced on the STN neurons oscillatory frequency (expressed in terms of frequency of max Power Spectral Density—PSD) and on the action selection efficacy of the model. The results of these analyses are shown in Figure 6.

The action selection mechanism efficacy was measured with the following statistics. For each test interval *I*1, *I*2, and *I*3 and for each group of M1 neurons belonging to one of the three channels, it was calculated the number of spikes with respect to the maximum number of spikes achievable for the lapse of time of the test interval (i.e., to the maximum activity). The resulting “three-test-intervals × three-channels” grid **G** was compared with a grid $\hat{\text{G}}$ of expected proportions. These expected proportions are (1, 0, 1) for the channel one (corresponding to a maximum activity in *I*1 and *I*3), (0, 1, 0) for the second channel (corresponding to a maximum activity in *I*2) and (0, 0, 0) for the third channel. The comparison measure *r*_*sel*_ consisted in determining the mean squared error (MSE) between the *K* = 9 groups of data:

(4) $$\begin{array}{r} r_{sel}=\frac{1}{k}\overset{K}{\underset{k}{\sum }}{\left(g_{k}-\hat{g_{k}}\right)}^{2} \end{array}$$

Figure 6 shows that it is necessary to have both low levels of dopamine baseline and low values of D2 receptor responsiveness to produce oscillations with a frequency in the range of 3–7 Hz, which is typical of tremor, and to have a low efficacy of the action selection mechanism. In particular, very low dopamine levels tend to impair action selection efficacy, especially if associated with low D2 responsiveness. Moreover, low dopamine baseline levels and low D2 receptors responsiveness strongly concur to cause the low frequency oscillations typical of tremor.

Overall, the data shown in Figures 4, 6 suggest the involvement of tonic dopamine dysfunction in the emergence of the behaviors typically exhibited by tremor patients.

## 4. Discussion

The data shown in Figures 3, 4 indicate that the dysregulation of phasic dopamine (timing and amplitude) impaires the action selection processes only if the difference between the two competing input signals (acting on two striatal channels) is small. In this case, the system is not able to switch between two subsequent movements showing an akinesia related to action sequencing. By contrast, if one of the two input signals is much larger than the other, as in the I1 interval, phasic dopamine dysfunctions are not sufficient to produce impairments in action selection. In this case, action impairments emerge only if also tonic dopamine is impaired.

The data illustrated in Figures 5, 6 suggest that low baseline dopamine levels and low responsiveness of dopamine D2 receptors reduce the efficacy of the action selection and produce oscillations at rest in the PD tremor range (around 3–7 Hz). These results are compatible with those observed in tremor patients (e.g., Rodriguez-Oroz et al., 2001) and with data showing that tonic dopamine levels are critical to regulate movement vigor (Mazzoni et al., 2007; Niv et al., 2007). Interestingly, the data shown in Figure 6 suggest that akinetic behavior could also be produced by low levels of dopamine. In particular, if low dopamine baseline levels add to a low responsiveness of D2 receptors the selection mechanism efficacy might be strongly impaired. In this condition, the system has a remarkable difficulty in initiating movements. This result agrees with empirical findings showing that some patients exhibiting tremor are also affected by akinesia (Zaidel et al., 2009; Zhang et al., 2015; Caligiore et al., 2016).

Overall these results support the hypothesis that akinesia and tremor could be produced by different dopaminergic dysfunctions. This perspective agrees with empirical data demonstrating the involvement of different neurobiological mechanisms for akinesia and tremor (Eidelberg et al., 1995; Brown et al., 2001; Mure et al., 2011). The model also suggests that the responsiveness of D2 receptors plays a critical role in the emergence of tremor (Figure 6). This view is supported by the literature underlying the critical role of D2 receptors to regulate the dopamine release in PD (Bolan et al., 2007; Hisahara and Shimohama, 2011). Overall, these results support the hypothesis that the akinetic form of PD involves different neuronal losses and different dysfunctional networks compared to the tremor dominant form. Further research is needed to validate this perspective. Indeed, it could for example be possible that clinical differences could be related to differences in phenotypic expression rather than with the neural system causes proposed here (Zaidel et al., 2009; Zhang et al., 2015).

The results presented in this work could be useful to devise new therapeutic actions for PD. Current drugs for PD treatment, in particular levodopa, have produce a generic effects on phasic and tonic dopamine and so are not suitable for a differential treatment of tremor and akinesia features (Connolly and Lang, 2014). The results obtained with the model suggest that different dopamine-related neural mechanisms cause tremor and akinesia and so it would be important to synthesize drugs that are able to specifically target those mechanisms.

### 4.1. Related Works

In the last decade, several computational models have been proposed to study PD (see Humphries et al. 2018 for a recent review). Most of these models reproduce critical anatomical and physiological features (Terman et al., 2002; Leblois, 2006; Kumar et al., 2011; Pavlides et al., 2012, 2015). Some works use more abstract mathematical models to study functional aspects of the basal ganglia-cortical loops (e.g., Holt and Netoff, 2014). These models typically focus on the functioning of the pallidal-subthalamic system, exploring the pathological mechanisms leading to abnormal oscillatory activity in a frequency range which is usually higher than the one characterizing parkinsonian tremor. In addition, although these models are capable of producing abnormal oscillations, their conclusions are limited by the partial reproduction of the basal ganglia-thalamo-cortical loop architecture. In this respect, the model proposed here demonstrates that PD features related to akinesia and tremor are the results of abnormal interactions between different brain areas, including basal ganglia nuclei, cortex, and thalamus. This system-level approach agrees with evidence showing that therapies based on brain stimulation can be effective even if applied to different districts of the basal ganglia-thalamo-cortical circuit (Johnson et al., 2008; Montgomery and Gale, 2008; Caligiore et al., 2016). Moreover, the system-level nature of the model has allowed the achievement of results that could not be obtained by reproducing only the functioning of the pallidal-subthalamic circuit. In particular, the model suggests that alongside this circuit also the input from cortices to striatum and to subthalamic nucleus are critical to produce tremor oscillations.

Among the models proposed in the literature, two are particularly relevant for the work presented here. The first one is the physiologically plausible model proposed by Humpries et al. to study the oscillatory properties of the basal ganglia circuitry under dopamine-depleted and dopamine-excessive conditions (Humphries et al., 2006). The model supports the critical role of the basal-ganglia action selection mechanism in the PD dysfunctions and also underlines the importance of system-level approaches to study PD. Moreover, it furnishes interesting predictions on the role of dopamine in the pallidal-subthalamic loop, showing that it is functionally decoupled by tonic dopamine under normal conditions and re-coupled by dopamine depletion.

These elements have been an important starting point for the design of the model presented here. However, there are some critical differences between the two models. First, the model of Humphries et al. does not reproduce the whole cortico-striatal-thalamo-cortical loops. This element, present in our model, is important to reproduce the system-level dynamics of the action selection mechanisms. As a consequence, in our model the abnormal oscillatory behavior characterizing tremor emerges as an effect of the dopamine dysregulation in the cortico-striatal-thalamo-cortical circuit. By contrast, the model of Humphries et al. is fed with an external oscillatory input injected into the cortex, rather than being intrinsically generated by the model on basis of its internal circuitry and mechanisms as it happens in brain. In this respect, the model is used to study how its circuits amplify or attenuate oscillatory perturbations when dopamine has different levels. The model is hence not used to show the genesis of tremor following dopamine dysregulation.

A second critical difference is that the model of Humphries et al. is primarily used to study the effects of tonic dopamine dysregulation but not those of phasic dopamine damage. Moreover, the model was used to show how alterations of the tonic dopamine levels reproduce data of slow (1 Hz) and γ-band (30–80 Hz) oscillatory phenomena reported in empirical works (MacKay, 1997; Brown et al., 2002). Instead, we implemented and manipulated both phasic and tonic dopamine, alongside the responsiveness of D2 receptors, to study how they might differently affect various features of akinesia and tremor.

The model proposed by Dovzhenok and Rubchinsky also represents an important precedent for the model presented here (Dovzhenok and Rubchinsky, 2012). This model, in agreement with converging empirical evidence, proposes a system-level mechanism supporting the idea that the basal ganglia-thalamo-cortical loop is the core oscillator at the origin of tremor. The authors show how the variation of the strength of dopamine-modulated connections in the basal ganglia-thalamo-cortical loop, equated to the decreased dopamine baseline levels in PD, leads to the occurrence of tremor-like burst oscillations. These oscillations are suppressed when the connections are modulated back to represent a higher level of dopamine, as it could happen following dopamine medication. The oscillations also cease when the basal ganglia-thalamo-cortical loop is broken, as it could happen in the case of ablative anti-parkinsonian surgery. Despite these relevant results, the authors implemented a very simplified model of the subthalamo-pallidal loop embedded into an abstract implementation of the basal ganglia-thalamo-cortical system. Moreover, the dopamine dysfunctions were reproduced in a rather indirect way by strengthening the subthalamo-pallidal loop. These features could limit the plausibility of the mechanisms explaining the target phenomena.

## 5. Conclusions

This article proposes a physiologically plausible model demonstrating that resting tremor could be primarily caused by low tonic dopamine levels, whereas akinesia could be due to both phasic and tonic dopamine impairments. In particular, the model predicts that phasic dopamine is mainly critical for producing sequences of actions, whereas tonic dopamine is principally involved in akinesia when starting a simple movement from rest. These features of the model could be critical to better characterize the different aspects of akinesia (Narabayashi, 1993; Thomas et al., 2003; Onofrj and Thomas, 2005) and represents a new viewpoint with respect to the widely studied role of phasic dopamine for learning in reinforcement-based contexts, according to which this signal could be related to reward expectation (Schultz, 1998; Dommett et al., 2005). Phasic dopamine activity associated with movement initiation has been observed in several works; however, the data collected in these studies comes primarily from studies in mice and, to date, similarly peri-movement dopamine bursts have not yet been convincingly demonstrated in primates. In addition, the sign, timing and learning-related properties of these activity are variable across studies (Jin and Costa, 2010; Dodson et al., 2016). In this respect, a recent study with mice has shown that during learning of a novel cue-reward association, there are midbrain dopaminergic neurons whose activity is related to the initiation of appetitive actions and dopaminergic neurons whose activity is associated with sensory cues that predict future reward. In both neural populations, excitation is modulated by expectation of reward (Coddington and Dudman, 2018). These issues could be addressed with future versions of the model.

The model proposed here represents the first step of a research agenda directed to study with a system-level perspective the mechanisms underlying different features of PD (Caligiore et al., 2016). To realize this agenda, the model will need to include other components and mechanisms. In particular, to characterize different forms of resting tremor the model might have to include cerebellar-thalamo-cortical circuits (Helmich et al., 2012; Wu and Hallett, 2013). To study the mechanisms for which levodopa medication might cause dyskinesias, reducing the effectiveness of the treatment, the model might have to reproduce the effects of serotonin modulation in the striatum (Reed et al., 2013; Politis and Niccolini, 2015). To investigate PD-related cognitive deficits the model might have to include the functioning of the prefrontal cortex (Frank, 2005; Guthrie et al., 2009). The reproduction of all these features of PD through suitable impairments of the same model is expected to lead to the progressive identification of the key system-level elements of brain that underlie the multifaceted manifestations of the disease.

## Author Contributions

DC and FM conceived the presented idea and developed the theory and the computational model. DC and FM designed the experiment. FM performed the simulations. DC, FM, and GB analyzed the data. All authors verified the analytical methods and discussed the results. DC wrote the paper. All authors gave feedback on the manuscript.

### Conflict of Interest Statement

The authors declare that the research was conducted in the absence of any commercial or financial relationships that could be construed as a potential conflict of interest.

## Footnotes

^1^https://www.python.org/

^2^https://github.com/francesco-mannella/nest-modmodule
